# Supplementary material for: Collaborative intelligence in AI: Evaluating the performance of a council of AIs on the USMLE
Source: PLOS Digit Health. 2025 Oct 9;4(10):e0000787. doi: 10.1371/journal.pdig.0000787 (PMC12510544; doi:10.1371/journal.pdig.0000787)
Supplement: S2 File — (ZIP) [file pdig.0000787.s002.zip › S2 Data Raw data files containing unprocessed question inputs and LLM outputs including transcripts of deliberations/STEP3_Question-84-1_Type-MC-NJ.docx]

**Facilitator** **[Round 1]:** A 37-year-old man comes to the emergency department because he has felt nauseated and light-headed for the past hour. Medical history is significant for esophageal varices secondary to alcohol-related cirrhosis and ascites treated with spironolactone. He drinks eight to ten alcoholic beverages daily. While you are obtaining additional history, the patient vomits a large volume of bright red blood and becomes difficult to arouse. Vital signs are temperature 36.0°C (96.8°F), pulse 110/min, respirations 12/min, and blood pressure 90/50 mm Hg. Following initiation of intravenous fluids, which of the following is the most appropriate immediate management? (A) Arrange for transjugular intrahepatic portal vein shunting (B) Begin intravenous vasopressin therapy (C) Do endotracheal intubation (D) Do upper endoscopy (E) Insert an esophageal tube for balloon tamponade

**AI_B:** 1) RESTATING THE QUESTION: We are presented with a case of a 37-year-old male who has been diagnosed with esophageal varices due to alcohol-related cirrhosis, against the backdrop of a history of heavy alcohol consumption. After initiation of intravenous fluids, we are asked to decide the most appropriate next step. 2) PLANNING: In reviewing his case, our main assumption is that his symptoms of nausea, light-headedness, and vomiting of bright red blood stem from his known esophageal varices, which commonly hemorrhage in the context of cirrhosis. If we consider that the patient's low blood pressure, tachycardia, and altered mental status might be due to the loss of blood volume, then our priority would be controlling the hemorrhage and managing the airway to prevent aspiration. 3) CLINICAL REASONING: Although management options like transjugular intrahepatic portal vein shunting, intravenous vasopressin therapy, upper endoscopy, or esophageal tube insertion for balloon tamponade might be valuable in certain circumstances, they either do not address the immediate issue at hand, or require an intact airway and hemodynamic stability. In such an unstable patient, endotracheal intubation seems most appropriate, as it not only secures the airway, but also can prevent the catastrophic complication of aspiration pneumonia. 4) CONCLUDING: Therefore, given the acute scenario of hematemesis, signs of hypotensive shock, and altered mental status, addressing his immediate risk of pulmonary aspiration via endotracheal intubation should take priority over further diagnostic or management steps. 5) RESPONSE: Hence, the most appropriate immediate management for this suffering patient would be endotracheal intubation (option C). The other options may be considered once he is stabilized.

**AI_E:** RESTATING THE QUESTION: This query pertains to a 37-year-old man with significant alcohol-related medical complications who has presented to an emergency department with a potentially critical gastrointestinal emergency, likely related to esophageal varices. The clinical picture suggests a potential variceal bleed, a life-threatening condition often associated with cirrhosis and alcohol abuse. The question is asking what the immediate priority intervention should be under these circumstances, with options being a transjugular intrahepatic porta-systemic shunt (TIPSS), intravenous vasopressin therapy, endotracheal intubation, upper endoscopy, or placement of an esophageal balloon tamponade. PLANNING: There are a few possibilities to consider here. TIPSS is a procedure used to decrease portal venous pressure and is usually reserved for patients who fail medical therapy or for those who continue to bleed despite therapy. Vasopressin contracts the smooth muscle in the splanchnic arterial bed, reducing portal venous inflow and pressure but can have severe side effects such as myocardial ischemia. Endotracheal intubation ensures airway protection, upper endoscopy is the direct method for diagnosing and potentially treating variceal bleeding, and esophageal balloon tamponade is a temporary measure often used while awaiting definitive therapy if initial measures are unsuccessful. CLINICAL REASONING: Given the patient's condition, the immediate priority is to maintain his airways, breathing, and circulation - ABCs. His hypotension, altered level of consciousness, and active hematemesis indicate the high risk of aspiration, which could lead to an aspiration pneumonia, a potentially life-threatening condition. Therefore, the priority is airway protection. CONCLUDING: As such, while interventions such as administering vasopressin, TIPSS, endoscopy, and esophageal balloon tamponade might all have their roles in the management of such a case, the immediate clinical priority considering the risk-benefit analysis and the necessity to follow ABCs principle would be to secure the airway to prevent aspiration of the vomited blood into his lung, as such aspiration could cause aspiration pneumonia or sudden and severe worsening of his respiratory status. RESPONSE: Therefore, the most appropriate immediate management under these circumstances is to (C) Do endotracheal intubation.

**AI_D:** 1) RESTATING THE QUESTION: A male patient with a history of alcoholic cirrhosis and esophageal varices comes into the ER with vomiting, lightheadedness, and nausea. Soon after his arrival, he vomits bright red blood and becomes less responsive. Given the urgent state of his condition, we seek to identify the most immediate and appropriate action to take to improve his condition. 2) PLANNING: Assumptions to consider are that his symptoms are due to his esophageal varices bleeding precipitated by his heavy alcoholic drinking, his low blood pressure and heart rate reflect a state of shock and/as severe blood loss, and the immediate priority is stabilizing the patient. If we question each assumption, the alternate possibilities are that the symptoms could be the resultant of other underlying, aggravated conditions, the vital signs reflect an entirely different cause, and focusing on a more immediate, but possibly temporary, intervention can buy us time for definitive treatment. In the context of this patient’s dangerous state, hemodynamic stabilization takes clinical precedence. 3) CLINICAL REASONING: Varices often bleed heavily due to high pressures in the portal vein system, and urgent control of bleeding combined with hemodynamic stabilization is paramount in such instances. Hence endotracheal intubation is prioritized to protect the airway and prevent aspiration of blood. This maneuver won't control the source of bleeding but it would decrease the chance of aspiration of blood in an altered consciousness patient, which is a immediate life-threatening situation. 4) CONCLUDING: While all of the listed options may be considered at some point in the management of massive esophageal hemorrhage, it's appropriate to first ensure that this already unstable patient does not go into respiratory arrest from aspirating on the fresh blood he is vomiting. Hence, intubation, in the present scenario, remains the highest-priority intervention. 5) RESPONSE: Given the presentation and medical history of the patient, the most appropriate immediate management to prevent aspiration of vomited blood and maintain airway protection would be to perform an endotracheal intubation (Choice C).

**AI_C:** RESTATING THE QUESTION: The scenario describes a 37-year-old man who has presented to the emergency department with nausea and light-headedness for the past hour. He has a medical history of esophageal varices due to alcohol-related cirrhosis and ascites, which is treated with spironolactone. during the consultation, the patient vomits a large quantity of fresh blood and becomes less responsive. His vital signs indicate tachycardia and hypotension. After starting intravenous fluids, the task is to determine the best immediate management action from choices that include transjugular intrahepatic portal vein shunting, intravenous vasopressin therapy, endotracheal intubation, upper endoscopy, and insertion of an esophageal tube for balloon tamponade. PLANNING: The current assumptions involve the patient's presentation (vomiting fresh blood) indicating active upper gastrointestinal bleeding, probably from his known esophageal varices. This situation is a medical emergency needing immediate treatment. Now, while all choices provided could play a role in managing such a patient at some point, the immediate step would usually be focused on securing airway, controlling bleeding, resuscitation, and diagnosis. Therefore the assumptions I would judge to be tested here is "Which of the following management options would be most critical to the patient's immediate survival?" CLINICAL REASONING: When managing a patient who vomits a large volume of blood and subsequently decreases their level of consciousness, our immediate concern should be airway protection. Endotracheal intubation would secure the airway to prevent aspiration pneumonia, which can result from inhaling vomited blood. Besides, having an impaired consciousness, indicated by the loss of arousability, also necessitates airway protection. CONCLUDING: This step is instrumental because aspiration of gastric contents into the lungs could potentially cause further morbidity and even mortality. As such, airway protection surpasses the need to control the source of bleeding, diagnose the source (even when it's quite clear from the history), or correct the underlying portal hypertension, which can be addressed after stabilizing the patient. RESPONSE: Given the urgency to protect the patient's airway due to the risk of aspiration, the most appropriate immediate management for this patient would be to perform endotracheal intubation (Choice C).

**AI_A:** RESTATING THE QUESTION: Our case revolves around a 37-year-old man who presents with symptoms of nausea and lightheadedness. His previous medical history involves esophageal varices attributed to cirrhosis caused by alcoholism and ascites. Despite his condition, he continues to consume a high amount of alcohol daily. In the middle of gathering further history, the patient vomits blood and becomes semi-responsive. His vital signs reveal slight fever, tachycardia, normal respirations, and hypotension. After administering intravenous fluids, we need to determine the next best step in treating this patient. The options presented are arranging for transjugular intrahepatic portal vein shunting, initiating IV vasopressin, performing endotracheal intubation, executing upper endoscopy, or inserting an esophageal tube for balloon tamponade. PLANNING: The primary concern here is the patient’s acute upper gastrointestinal bleeding, most likely from his known esophageal varices. It is also crucial to acknowledge his alcohol abuse as it continues to compound his liver damage, making the overall situation more complex. The patient's current condition requires immediate management to stop the bleeding, stabilize his vitals, and prevent aspiration pneumonia, which can result from the inhalation of vomit into the lungs. CLINICAL REASONING: This patient's semi-responsive state, vomiting of blood (which suggests ongoing hemorrhage), and low blood pressure (indicative of hypovolemic shock) present an imminent threat to life. Left unmanaged, this can lead to serious complications including aspiration pneumonia, hypoxemia, and further reduction in blood volume, leading to cardiovascular collapse. Management must therefore prioritize protecting the patient's airway, followed by stopping the bleeding. All the given options can contribute to managing the patient's state but need to be prioritized in terms of immediate and subsequent actions. CONCLUDING: The clinical priority in such cases is always airway management before proceeding to endoscopic or pharmacologic therapy. Protecting the airway limits the potential for aspiration pneumonia, which could complicate the patient's picture and prognosis. Once the airway is secure, the source of bleeding can then be managed directly via endoscopy or indirectly with medications or procedures. RESPONSE: Thus, the most appropriate immediate management in this case is (C) endotracheal intubation. This procedure will protect the airway from aspiration of blood and other stomach contents. Management of the bleeding can follow after the airway is secure.

**Facilitator** The consensus statement synthesizes the responses of AI_A, AI_B, AI_C, AI_D, and AI_E as follows: The case involves a 37-year-old man with a history of alcohol-related cirrhosis and esophageal varices, who presents with symptoms of nausea, lightheadedness, and vomiting of bright red blood. His condition deteriorates as he becomes less responsive, indicating an altered level of consciousness. His vital signs reveal tachycardia and hypotension, suggesting a state of shock, likely due to significant blood loss. The immediate concern in this scenario is the patient's acute upper gastrointestinal bleeding, most likely from his known esophageal varices, and the potential risk of aspiration due to his altered mental status. Given the urgency of the situation, the immediate priority is to secure the patient's airway to prevent aspiration of vomited blood, which could lead to aspiration pneumonia, a potentially life-threatening condition. This is in line with the principle of maintaining airways, breathing, and circulation (ABCs) in emergency medicine. Therefore, the most appropriate immediate management in this case is endotracheal intubation. While other management options such as transjugular intrahepatic portal vein shunting, intravenous vasopressin therapy, upper endoscopy, and insertion of an esophageal tube for balloon tamponade may be considered at later stages, they either do not address the immediate issue at hand or require an intact airway and hemodynamic stability. These interventions can be pursued once the patient's airway is secure and his condition is stabilized.
